# Determinants of hypertension in a young adult Ugandan population in epidemiological transition—the MEPI-CVD survey

**DOI:** 10.1186/s12889-015-2146-y

**Published:** 2015-08-28

**Authors:** James Kayima, Joaniter Nankabirwa, Isaac Sinabulya, Jane Nakibuuka, Xiaofeng Zhu, Mahboob Rahman, Christopher T. Longenecker, Achilles Katamba, Harriet Mayanja-Kizza, Moses R. Kamya

**Affiliations:** Department of Medicine, Makerere University College of Health Sciences, P.O. Box 7072, Kampala, Uganda; Clinical Epidemiology Unit, Makerere University College of Health Sciences, Kampala, Uganda; Department of Epidemiology, Case Western Reserve University School of Medicine Cleveland, Cleveland, OH USA; Clinical Hypertension Program, University Hospitals Case Medical Center, Cleveland, OH USA; Harrington Heart and Vascular Institute, University Hospitals Case Medical Center, Cleveland, OH USA

## Abstract

**Background:**

High blood pressure is the principal risk factor for stroke, heart failure and kidney failure in the young population in Africa. Control of hypertension is associated with a larger reduction in morbidity and mortality in younger populations compared with the elderly; however, blood pressure control efforts in the young are hampered by scarcity of data on prevalence and factors influencing awareness, treatment and control of hypertension. We aimed to describe the prevalence of prehypertension and hypertension among young adults in a peri-urban district of Uganda and the factors associated with occurrence of hypertension in this population.

**Methods:**

This cross-sectional study was conducted between August, 2012 and May 2013 in Wakiso district, a suburban district that that encircles Kampala, Uganda’s capital city. We collected data on socio-demographic characteristics and hypertension status using a modified STEPs questionnaire from 3685 subjects aged 18–40 years selected by multistage cluster sampling. Blood pressure and anthropometric measurements were performed using standardized protocols. Fasting blood sugar and HIV status were determined using a venous blood sample. Association between hypertension status and various biosocial factors was assessed using logistic regression.

**Results:**

The overall prevalence of hypertension was 15 % (95 % CI 14.2 – 19.6) and 40 % were pre-hypertensive. Among the 553 hypertensive participants, 76 (13.7 %) were aware of their diagnosis and all these participants had initiated therapy with target blood pressure control attained in 20 % of treated subjects. Hypertension was significantly associated with the older age-group, male sex and obesity. There was a significantly lower prevalence of hypertension among participants with HIV OR 0.6 (95 % CI 0.4–0.8, P = 0.007).

**Conclusion:**

There is a high prevalence of high blood pressure in this young periurban population of Uganda with sub-optimal diagnosis and control. There is previously undocumented high rate of treatment, a unique finding that may be exploited to drive efforts to control hypertension. Specific programs for early diagnosis and treatment of hypertension among the young should be developed to improve control of hypertension. The relationship between HIV infection and blood pressure requires further clarification by longitudinal studies.

**Electronic supplementary material:**

The online version of this article (doi:10.1186/s12889-015-2146-y) contains supplementary material, which is available to authorized users.

## Background

Cardiovascular disease (CVD) is the leading cause of death in developing countries contributing to 30 % of all global deaths [1]. The age-specific mortality rates from cardiovascular complications are much higher in younger age groups for both sexes in Africa than in the developed world [2, 3]. Hypertension is a driver of the cardiovascular disease epidemic with 16.5 % of all global deaths attributable to high blood pressure [4]. In sub-Saharan Africa, high blood pressure is the leading risk factor for heart failure, stroke and kidney failure, which characteristically occur at younger ages [5–7]. The economic impact of hypertension in Africa is felt directly by the individuals and the health care system through the high costs incurred in treating complications; and indirectly through the loss of household incomes due to disability and death of these younger adults [8].

Hypertension is a modifiable risk factor whose treatment and control has been partly responsible for the dramatic decline in morbidity and mortality in developed countries over the last thirty years [9, 10]. Blood pressure levels from young adulthood predict the incidence of future cardiovascular events and more significant improvements in mortality and morbidity from blood pressure control have been reported among younger subjects when compared to older ones [11, 12]. In Africa, suboptimal blood pressure control remains challenging—a recent systematic review showed varied prevalence and excessively low awareness, treatment and control of hypertension [13]. There are limited data on the prevalence of high blood pressure and its risk factors among the young, whereas this is the population that could derive the most benefit from control efforts. In addition, the influence of novel risk factors for CVD such as HIV infection has not been well described.

In Uganda, most of the available data on hypertension has been hospital-based [14]. The few community studies that have been conducted pertain mainly elderly population and have demonstrated an increasing burden of hypertension and its risk factors associated with low levels of awareness, low treatment coverage and poor control [15–17]. The small sample sizes of generally older adults from rural populations has limited generalizability and hindered the formulation of a comprehensive policy for prevention and control of hypertension among the young. In this study, we investigated the prevalence and factors associated with high blood pressure among younger adults in a peri-urban district of Uganda under the Medical Education Partnership Initiative- Cardiovascular Disease Survey (MEPI-CVD Survey).

## Methods

### Community survey

This cross-sectional study was conducted between August, 2012 and May 2013 among adults >18 years in Wakiso district, Central Uganda. Wakiso district encircles Kampala, the capital of Uganda, and is the most populous district in the country with an estimated population of 2 million according to the 2014 census [18]. This district is undergoing rapid urbanisation due to its proximity with the capital city yet it still has large areas of rural dwellings; a classic case of the epidemiological transition. Wakiso has seven health sub districts (HSD) each with a health center. In the hierarchy of district health service organization the HSD is a tier lower than the district.

Multi-stage sampling was used to select the HSD of Wakiso district in which the survey was carried out. First, the HSDs were stratified into rural and suburban and then one sub-county was chosen from each stratum by simple random sampling (Nansana town council for the urban and Busukuma for the rural). Nansana has 23 villages, while Busukuma has 48 villages. All the households and other key features in these villages were enumerated and mapped using hand-held eTrex global positioning system (GPS) receivers (Garmin Ltd. Olathe, KS) to generate a sampling frame. A household was defined as any single permanent or semi-permanent dwelling acting as the primary residence for a person or group of people that generally cook and eat together. The mapping returned 26,757 periurban households and 13,091 rural households which were included in the randomization of sample.

Prior to the survey day, mobilization teams of village health team (VHT) members visited the study villages in order to raise awareness of the study. The mobilization of study villages focused on engaging with participants at both a community and individual level, with the aim of achieving and maintaining high levels participation. This process involved meeting local leaders with letters detailing the reason for the study and the duration of planned work; holding a series of open community meetings to increase general awareness of the study and notifying study participants about the arrival of the study team to ensure maximum participant availability.

A list of households to be approached for each site was randomly computer generated from the enumeration database. Study personnel visited the selected households in sequential order to identify houses with at least one adult resident to include in the study. Residents who were not home during the initial contact were re-visited on three other occasions before excluding them from the study. In each household, one adult (>18 years old) was randomly selected to participate in the survey. This person was briefed about the study in the appropriate language and asked to attend a centrally placed research clinic the following day after at least an eight hour fast. Each participant was given a numbered card for identification on the survey day. Households were included if: 1) they had an adult aged 18 years or older, and 2) the selected adult was willing to provide written informed consent. Households were excluded if: 1) no adult resident was at home on more than 3 visits or 2) if the household was vacant.

On the survey day, written consent from participants in the study was sought and a survey instrument, based on the World Health Organization (WHO) modified expanded STEPs questionnaire [19] was administered by research assistants. The survey instrument has three levels. Level 1 contains the core or “minimum set” of self-report measures including demographic data (age, sex, and address), tobacco and alcohol consumption, exercise, smoke exposure, socio-economic status based on housing characteristics, family history and personal history of hypertension including the awareness of hypertensive status, diabetes and dyslipidemia and history of treatment for these conditions if any. Level 2 contains simple physical measurements including height, weight, waist circumference, and blood pressure. Level 3 assess biochemical measurements which involves collecting venous blood collection for biochemical assessments including fasting glucose and lipids. The survey instrument was modified to gather information about other non-traditional risk factors for cardiovascular disease such as HIV infection.

### Blood pressure

Blood pressure and heart rate were measured with an Omron automated sphygmomanometer model HEM-907 whose accuracy has been validated [20]. Participants were asked to refrain from smoking cigarettes and drinking alcohol or caffeinated beverages for at least 30 min prior to examination. The blood pressure in the left arm was measured after resting for at least five minutes. The blood pressure was taken in the sitting position, legs uncrossed, with the arm resting on a table and the ante-cubital fossa at the level of the lower sternum. Two arm cuffs that fit arm circumferences 9–13 in. and 13–17 in. were used in the process. Three blood pressure readings were measured to the nearest mmHg three minutes apart and the mean of the closest two values were used for analyses [21]. Height was measured to the nearest 0.1 cm with a SECA 214 portable stadiometer while the subjects stood barefoot on the centre of the base with their back to the stadiometer. The weight was measured to the nearest 0.1 kg with a SECA 762 scale. These recordings were used to derive the body-mass index.

### Laboratory measurements

Fasting venous blood samples were collected by standardized aseptic technique and were transported to a laboratory for analysis within two hours of collection. Glucose was measured immediately on site by a glucometer. Total cholesterol, high-density lipoprotein, and triglyceride concentration were measured using Cobas c111 automated chemistry analyzer (Roche Diagnostics GmbH, Mannhein, Germany). The HIV status was determined using the rapid testing algorithm which included the Determine HIV-1/2 assay (Abbott Laboratories, Illinois, United States of America) for screening and the HIV-1/2 STAT-PAK Dipstick assay (Chembio Diagnostic Systems Inc., New York, USA) for confirmation of HIV status.

### Statistical analysis

Data were collected using hand-held computers programmed to include range checks, structure checks and internal consistency checks. For this analysis we included all participants between the ages of 18–40 years, stratified by younger (18–29) and older (30–40) groups. The outcomes of interest were: (1) prehypertension defined as systolic blood pressure between 120 and 139 mmHg and/or diastolic between 80 and 89 mmHg; (2) hypertension which was defined as a systolic blood pressure greater than 140 mmHg and/or a diastolic blood pressure greater than 90 mmHg or treatment with anti-hypertensive medication; (3) severity of hypertension using categories defined by the United States Seventh Joint National Committee on Detection, Evaluation and Treatment of Hypertension (JNC-VII) [22]; (4) awareness status defined as participants having been told by a health worker that they had hypertension; (5) treatment status defined as any prescribed treatment for high blood pressure including diet, exercise, weight loss, smoking cessation and pharmacological therapy; and (6) blood pressure control defined as treatment to a target systolic blood pressure of less than 140 mmHg and diastolic less than 90 mmHg.

Categorical variables were summarized using frequency (%), while continuous variables were summarized using means. Univariable associations between hypertension and potential risk factors were assessed using logistic regression and all variables demonstrating an association at a p < 0.2 significance level were candidates for inclusion in the multivariable logistic regression model. Logical model building using both forward and backward elimination was used to generate a minimum adequate model in a stepwise fashion using a p < 0.05 significance level for inclusion in the model. All statistical analyses were carried out using Stata version 12.0 software (STATA Corporation, College Station, TX).

### Ethical approval

The survey protocol was approved by the Makerere University School of Medicine Research and Ethics Committee and the Uganda National Council of Science and Technology. Written informed consent was obtained from all survey participants. Participants diagnosed with clinical conditions including diabetes, hypertension, and HIV infection were given initial treatment by our clinical team and referred to the nearest health sub district center for further follow up.

## Results

A total of 3920 participants between the ages of 18 and 40 years were screened during the survey. Complete data on hypertension and its determinants was obtained from 3685 (94 %) participants who were included in the analysis. Table 1 summarizes the characteristics of the study participants. The majority of the participants (69 %) were female. Seventy-eight percent were from the periurban Nansana health sub-district. The median age of the participants was 27 years; 62 % were in the young age-group (18–29 years). Normal BMI was registered in 59 % of the study participants while 23 % were overweight and 12 % were obese. The HIV prevalence was 9.1 % of our study population compared to an estimated nationwide prevalence of 7.3 % [23]. More baseline characteristics are shown in Additional file 1: Table S1.

**Table 1:** Socio-demographic characteristics of the study participants aged 18–40 years in MEPI- CVD survey

Variable	n (%)
Age in years (n, %)	
18–29	2,270 (61.6)
30–40	1,415 (38.4)
Sex	
Male	1,158 (31.4)
Female	2,527 (68.6)
Sub-county (n, %)	
Nansana Town Council (peri-urban)	2,866 (77.8)
Busukuma (rural)	819 (22.2)
Social economic status	
Poorest	819 (22.2)
Poor	1,297 (35.2)
Less poor	604 (16.4)
Least poor	965 (26.2)
Smoking	
Never smoked	3,393 (92.1)
Previously smoked	117 (3.2)
Currently smoking	173 (4.7)
Alcohol	
No intake	3,258 (88.5)
Mild intake	114 (3.1)
Moderate intake	219 (5.9)
Heavy intake	91 (2.5)
Physical activity	
Active	3,485 (94.9)
Inactive	189 (5.1)
BMI	
Underweight	201 (5.5)
Normal	2,174 (59.3)
Overweight	847 (23.1)
Obese	447 (12.2)
Family history of high blood pressure (n, %)	
No	2,775 (75.5)
Yes	903 (24.5)
History of diabetes (n, %)	
No	3,666 (99.7)
Yes	12 (0.3)
HIV status	
Negative	3,351 (90.9)
Positive	334 (9.1)
Triglycerides (mg/dl)	
<150	2,961 (80.8)
150–199	319 (8.7)
200–499	331 (9.0)
≥500	56 (1.5)
Non HDL cholesterol	
Ideal	2,904 (79.3)
Borderline	181 (4.9)
High	575 (15.7)

### Prevalence and distribution of hypertension and prehypertension

**Fig. 1:**
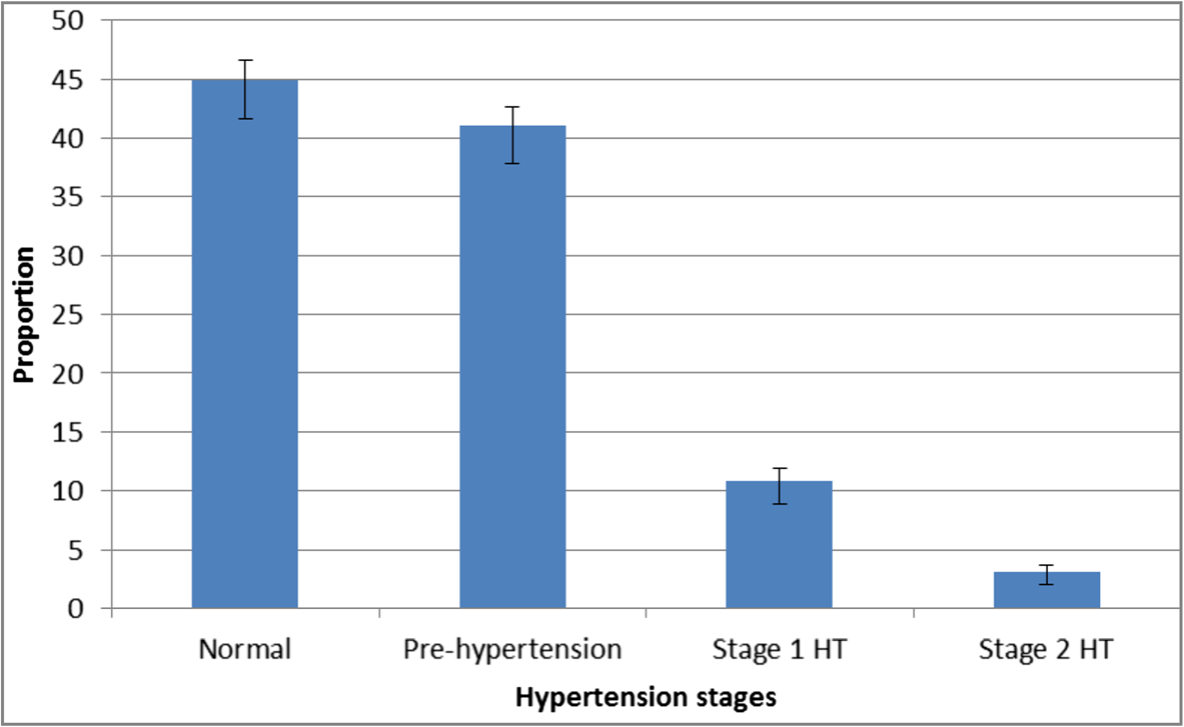
Prevalence of different stages of hypertension among adults (age 18–40 years) in Wakiso district central Uganda. Error bars represent standard deviation. HT: hypertension

**Fig. 2:**
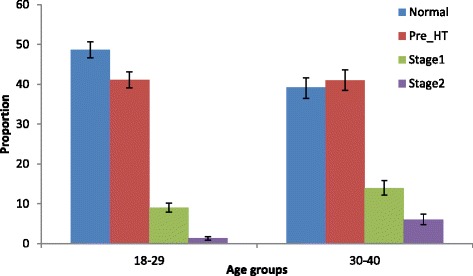
Hypertension classified using the JNC VII criteria by age-group among adults (age 18–40 years) in Wakiso district Central Uganda. Error bars represent standard deviation

The overall prevalence (95 % CI) of hypertension in this population was 15 % (95 % CI 14.2 – 19.6 %) %. Figure 1 summarizes the prevalence of different stages of hypertension among the study participants using JNC VII classification according to both systolic and diastolic blood pressure. Of all the study participants, 114 (3.1 %) had severe hypertension (stage 2) and 402 (11 %) had stage 1 hypertension. Only 45 % of participants had a normal blood pressure and 41 % had prehypertension. Figure 2 shows the stages of hypertension by age group. The prevalence of prehypertension was similar in both age groups; however, hypertension was more prevalent in the older age group (10.8 % vs 21.6 %, p = 0.001).

### Factors associated with hypertension

In this survey population, female participants were less likely to be hypertensive compared to men OR 0.7 (95 % CI 0.6–0.8, P < 0.001). See Additional file 2: Table S2. There was a trend towards increased prevalence of hypertension among participants with higher socioeconomic status which was not significant after multivariable adjustment. There was also a non-statistically significant trend towards increased hypertension prevalence among smokers and those with heavy alcohol intake. The participants’ diabetes status (history of diabetes and diagnosis of diabetes during the survey) was positively associated with hypertensive status 3.1 (0.9–10.8, p = 0.007). Participants with HIV infection were less likely to be hypertensive in this study population OR 0.6 (95 % CI 0.4–0.8, p = 0.007). There was a graded increase in hypertension prevalence among the overweight and obese participants in the survey.

### Awareness, treatment and control

**Fig. 3:**
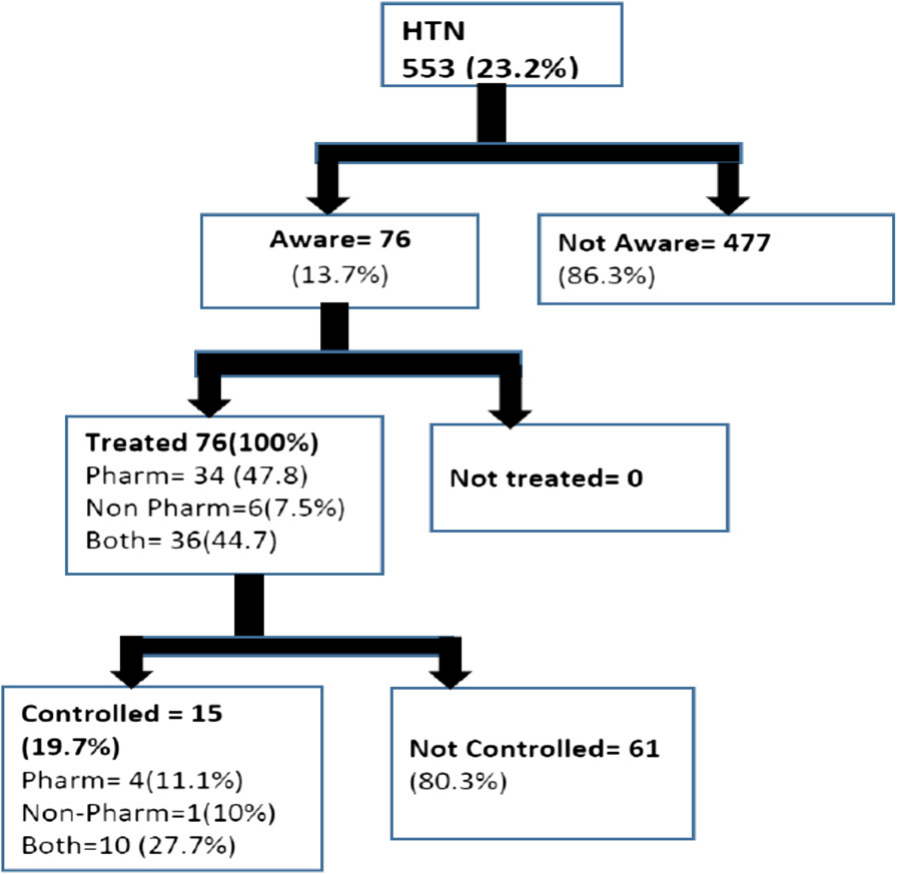
Awareness, treatment and control of hypertension among adults (age 18–40 years) in Wakiso district, central Uganda. HTN: hypertension; Pharm: pharmacological means of treatment; Non–pharm: non-pharmacological means of treatment

**Fig. 4:**
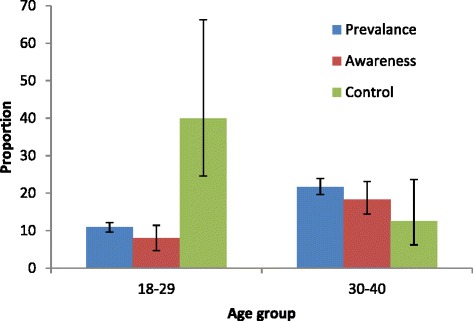
Prevalence, awareness and control by age-group among adults (age 18–40 years) in Wakiso district Central Uganda. Error bars represent standard deviations

Figure 3 shows the proportions of awareness, treatment and control among the hypertensive study participants. Among the 553 hypertensive participants, 76 (13.7 %) were aware of their condition and all were on some form of therapy. Most of the participants on treatment (47.7 %) used only pharmacological means for control of blood pressure, while the minority used on the non-pharmacological means including exercise, diet and smoking cessation. The participants who used both pharmacological and non-pharmacological means of treatment achieved better control of hypertension with more than a quarter of them attaining target blood pressure. Only about 10 % of those using pharmacological means only were treated to target. Overall, control was achieved in a fifth of the population. Figure 4 shows treatment, awareness and control of hypertension among the study population by age-group. The awareness and treatment of hypertension (18 %) was higher in the older age group compared to the younger group (8.1 %); however, the younger age group had better control of hypertension with 40 % of all treated hypertensive controlled compared to 13 % in the older age-group.

## Discussion

In order to develop a more coherent policy for the control of hypertension and for the mitigation of its adverse outcomes in Uganda, it is important to have a more precise estimate of the prevalence of the condition and to determine the factors that are associated with it. Our results demonstrate that high blood pressure is highly prevalent in this young population with almost a sixth of the study population being classified as hypertensive. The prevalence of hypertension in our study population is comparable to that of other young populations in Africa in periurban areas which has ranged from 14.5 % in Ethiopia to 23 % in Tanzania [24, 25]. Comparison with recent studies in Uganda was difficult as most of them have considered elderly populations with few young participants [15]. Other studies such as Wamala et al. and Musinguzi et al. have focused mainly on rural districts in Western and Central Uganda, respectively, with only a few trading centres within the study sites considered periurban [16, 17]. While these studies did not intently focus on young subjects, the prevalence of hypertension in their younger subjects at 16.7 % was strikingly similar to that in our population. The young subjects in this survey may be more representative of the Ugandan adult population, which according to the national census is fairly young with a median age of 15.5 years [18]. Equally important is the finding of high prevalence of prehypertension in this survey considering that there is good evidence that prehypertension in young individuals significantly raises cardiovascular risk [26–28]. The increasing prevalence of hypertension and prehypertension in Africa has been blamed on acculturation- the adoption of Western ways of living in places with rapid urbanisation. Dietary trends toward higher sodium and lower potassium are widespread throughout rural and urban areas of Africa. Our study is a classic example of a population undergoing epidemiological transition due to rapid urbanization. Because appropriate management of the complications of hypertension may not be attainable in some African settings due to limited resources, primordial methods of prevention that stress lifestyle changes ought to be directed towards young adults to improve long-term outcomes.

Despite the high prevalence of hypertension, less than a fifth of the subjects with hypertension were aware of their condition. The finding of sub-optimal diagnosis in our survey is consistent with observations in other developing countries outside Africa, but also in the developed world where diagnosis of hypertension in the young population is usually low [29]. In developed countries physician inertia and health disparities have been blamed for the low levels of diagnosis while in Africa weak health systems related to both structure and financing are thought to contribute to this situation [30]. Efforts to improve the detection of hypertension should focus on identifying younger subjects with or at-risk for hypertension especially because there is evidence that prevention and treatment of hypertension in young people is associated with greater risk reductions than in the elderly [12]. In many randomised controlled trials in the developed countries, community screening has led to an improved detection of hypertension [31].

All participants who were aware of their hypertensive status were on some form of treatment with control to target achieved in only a fifth of those treated. A multi-faceted approach to therapy using both antihypertensive drugs and lifestyle measures delivered better control of hypertension. The finding of high treatment rates is an uncommon finding in this African setting with treatment rates in other parts of Africa ranging from 5 % in a rural Nigerian community to 91.2 % in urban North African populations [13]. The exceptional levels of hypertension treatment may be explained by improved public-health messaging on the dangers of hypertension from the Uganda Ministry of Health in Wakiso District, which is often the first recipient of health promotion efforts [32]. The poor control of hypertension despite treatment is comparable to that in many African countries and may be related to health system related deficiencies which are common in African settings [33, 34]. Because control to target is the ultimate predictor of outcomes for hypertensive patients, these findings call for a concerted effort to improve these statistics in sub-Saharan Africa. The utilization of both drugs and lifestyle modification to treat hypertension is now recommended as an approach to deliver more sustainable control of blood pressure in young individuals [35]. In addition, leveraging chronic disease care models from the highly successful HIV/AIDS control efforts has been proposed as a health system alternative for non-communicable disease treatment and control. Among other components, these models have included task-shifting, health systems strengthening and support for adherence and retention [36].

In our study, hypertension was more prevalent in the older population, consistent with the known epidemiology globally and in other parts of Africa [15, 16, 37]. While age related increase in blood pressure is currently considered a universal feature of human aging, the trajectory of this increase has been shown in longitudinal studies to be steeper among younger subjects in settings undergoing rapid urbanization [38, 39]. Recent studies have revealed that this age-related rise in blood pressure seems to occur much later in subjects with better cardiorespiratory fitness stressing the necessity of primary prevention of hypertension [38]. In addition to age related differences in hypertension prevalence, gender-related differences were prominent in our study, with the men having a significantly higher prevalence. Gender differences in hypertension have been studied extensively. It is well-recognized that young men are more likely to develop high-blood pressure and have poorly controlled hypertension than premenopausal women, a dynamic that has been attributed to androgen mediated abnormalities in pressure natriuresis [40, 41]. However, large meta-analyses of hypertension treatment trials have failed to document gender differences in response to antihypertensive medication meaning that the poor control of hypertension documented in men is probably due to socio-economic and cultural factors [42]. This finding calls for the formulation of gender specific programs to address the gender differences in access to and utilization of health care facilities for the control of hypertension.

The HIV infection prevalence in our study population was higher than the national prevalence of 7.4 % [23]. These HIV infected participants were less likely to be hypertensive than the uninfected participants. The results of studies on effect of HIV on hypertension prevalence in the Africa have been inconsistent. There have been suggestions that antiretroviral therapy has not only increased life expectancies for HIV-infected adults but has also put them at greater risk for central obesity and hypertension [43, 44]. However, most of these studies have been in HIV infected cohorts with no comparable group. Recent studies in African settings that included HIV-uninfected comparison groups are in agreement with our findings of a lower prevalence of hypertension in HIV infected individuals, regardless of whether they were taking anti-retroviral drugs or not [45–47]. Dysregulation of sympathetic nervous system and hypoadrenalism have been suggested to account for the lower blood pressures found in HIV infected patients and may be associated with increased mortality [48–50]. Because we lacked data on current CD4+ T-cell count or HIV-1 viral load, our study was unable to explore covariates that may have been associated with low or high blood pressure in the HIV-infected population.

Over a third of our study population was overweight or obese which were significantly associated with hypertension as expected. The positive relationship between body weight and blood pressure has been reported in longitudinal studies and has been replicated in other rapidly urbanizing settings in sub-Saharan Africa [51, 52]. The growing obesity epidemic in sub-Saharan Africa has been largely attributed to increasing consumption of western-style diets high in sugar and fat [53]. However, cultural perceptions that value heavier body weight as a sign of wellbeing and wealth cannot be underestimated [54, 55]. To be able to successfully confront obesity-related hypertension in Africa, education on the consequences of obesity should be an integral part of control efforts.

Some strengths of this survey warrant to be mentioned. To our knowledge this is the first study that has focused on hypertension in young adults in epidemiological transition in East Africa. Our findings are potentially generalizable to similar age groups in similar rapidly urbanizing settings. Our survey had a relatively large sample size, employed rigorous epidemiologic methods including training of the research assistants and standardized instruments and had high response rate which contributed to strength of the study. Certain limitations should be considered in the interpretation of the study results. First, known history of hypertension was self-reported and therefore prone to recall bias. This could have led to overestimation of rates of awareness and control of hypertension; however, prior studies have validated self-report as a way of determining the diagnosis of hypertension [56]. Secondly, blood pressure was measured at a single point in time because of the difficulty of multiple measurements in a survey of this magnitude; however, the method of blood pressure measurement was highly standardized and performed in triplicate after an adequate rest period. Finally, data on medication adherence were not collected as a possible predictor of hypertension control.

## Conclusions

Overall, the findings of this survey reveal a high prevalence of elevated blood pressure (prehypertension and hypertension) in this young periurban population with suboptimal levels of diagnosis and control. We documented a high rate of treatment, a unique finding that may be exploited to drive efforts to control hypertension. Hypertension was significantly associated with the older age group, male sex and being overweight or obese while HIV infection was negatively associated with high blood pressure. Specific programs for early diagnosis and treatment of hypertension among the young should be developed to improve control of hypertension. The relationship between hypertension and HIV infection needs further clarification in longitudinal studies.

## Additional files

Additional file 1: Table S1. Additional baseline characteristics of the survey population in Wakiso district, Central Uganda. (PDF 153 kb) Additional file 2: Table S2. Factors associated with hypertension among adults (age 18–40 years) in Wakiso district, Central Uganda. (PDF 175 kb)

## Abbreviations

CVD: Cardiovascular disease
MEPI-CVD: Medical education partnership on cardiovascular disease
VHT: Village health team
HSD: Health sub-district
