# Supplementary material for: Determinants of hypertension in a young adult Ugandan population in epidemiological transition—the MEPI-CVD survey
Source: BMC Public Health. 2015 Aug 28;15:830. doi: 10.1186/s12889-015-2146-y (PMC4552375; doi:10.1186/s12889-015-2146-y)
Supplement: Additional file 1: Table S1. — Additional baseline characteristics of the survey population in Wakiso district, Central Uganda. (PDF 153 kb) [file 12889_2015_2146_MOESM1_ESM.pdf]

Additional Table 1: Additional baseline characteristics of the study population

Variable	n (%)
Blood pressure	
Mean systolic (SD)	120.3 (15.3)
Mean diastolic	76.7 (10.4)
Hypertensive	
No	3,122 (84.9)
Yes	552 (15.1)
Mean blood glucose (SD)	5.8 (23.4)
Median age (IQR)	27 (23 -32)
Mean weight (SD)	62.8 (12.4)
Mean waist circumference (SD)	78.6 (11.2)
Mean hip circumference (SD)	96.6 (11.1)
Waist hip ratio	
Normal	2,941 (80.1)
Obese	732 (19.9)
Mean height (m, SD)	1.6 (0.1)
Median Triglycerides (mg/dl, IQR)	85.9 (58.5 – 130.2)
Median LDL (gm./dl, IQR)	36.7 (18.2 – 91.6)
Mean HDL (gm/dl, SD)	42.2 (17.6)
Total Cholesterol gm./dl (Median, IQR)	116.0 (88.9 – 169.4)

IQR (Interquartile range)

SD (Standard deviation)
