# Supplementary material for: Determinants of hypertension in a young adult Ugandan population in epidemiological transition—the MEPI-CVD survey
Source: BMC Public Health. 2015 Aug 28;15:830. doi: 10.1186/s12889-015-2146-y (PMC4552375; doi:10.1186/s12889-015-2146-y)
Supplement: Additional file 2: Table S2. — Factors associated with hypertension among adults (age 18–40 years) in Wakiso district, Central Uganda. (PDF 175 kb) [file 12889_2015_2146_MOESM2_ESM.pdf]

Additional Table 2: Factors associated with hypertension among adults (age18-40 years) in Wakiso district, Central Uganda

Variable	n/N	Crude OR (95% CI)	p-value	Adjusted OR (95% CI)	p-value
Age-group in years					
18 – 29*	247/2,270	1		1	
30 - 40	306/1,415	2.3 (1.9 – 2.7)	<0.0001	**2.0 (1.6 – 2.5)**	**<0.001**
Sex					
Male*	211/1,158	1		1	
Female	324/2,527	0.7 (0.6 – 0.8)	<0.0001	**0.6 (0.5 – 0.7)**	**<0.001**
Sub-county					
Nansana Town Council*	432/2,866	1			
Busukuma	121/819	0.98 (0.8 – 1.2)	0.833		
Social economic status					
Poorest*	109/819	1			
Poor	176/1,297	1.02 (0.8 – 1.3)	0.864		
Less poor	92/604	1.2 (0.8 – 1.6)	0.304		
Least poor	176/965	1.5 (1.1 – 1.9)	0.005		
Family history of hypertension					
No*	391/2,775	1		1	
Yes	162/903	1.3 (1.1 – 1.6)	0.005	1.2 (0.9 – 1.4)	0.144
Smoking					
Never smoked*	493/3,393	1			
Previously smoked	21/117	1.3 (0.8 – 2.1)	0.305		
Currently smoking	39/172	1.7 (1.2 – 2.5)	0.004		
Alcohol					
No intake*	482/3,258	1		1	
Mild intake	13/114	0.7 (0.4 – 1.3)	0.316	0.7 (0.4 – 1.3)	0.252
Moderate intake	34/219	1.1 (0.7 – 1.5)	0.768	0.9 (0.6 – 1.3)	0.513
Heavy intake	24/91	2.1 (1.3 – 3.3)	0.003	1.6 (0.9 – 2.7)	0.061
Physical activity					

Active*	528/3,485	1		1	
Inactive	24/189	0.8 (0.5 – 1.3)	0.359	0.7 (0.4 – 1.1)	0.127
BMI					
Underweight*	23/201	1		1	
Normal	280/2174	1.1 (0.7 – 1.8)	0.559	1.2 (0.8 – 1.9)	0.398
Overweight	141/847	1.5 (0.9 – 2.5)	0.070	**1.6 (1.01 – 2.7)**	**0.044**
Obese	107/447	2.4 (1.5 – 3.9)	<0.0001	**2.6 (1.5 – 4.3)**	**<0.001**
History of diabetes (n, %)					
No*	547/3,666	1		1	
Yes	6/12	5.7 (1.8 – 17.7)	0.003	**3.1 (0.9 – 10.8)**	**0.007**
Fasting blood glucose					
Normal*	430/2,844	1		1	
Impaired	89/687	0.8 (0.7 – 1.1)	0.151	0.8 (0.6 – 1.03)	0.092
Diabetic	23/94	1.8 (1.1 – 2.9)	0.015	1.5 (0.9 – 2.5)	0.102
HIV					
No*	521/3,351	1		1	
Yes	32/334	0.6 (0.4 – 0.8)	0.004	**0.6 (0.4 – 0.9)**	**0.007**
Non HDL cholesterol					
Ideal*	452/2,904	1			
Borderline	23/181	0.8 (0.5 – 1.2)	0.302		
High	73/575	0.8 (0.6 – 1.02)	0.080		

*Reference group
